# ATL-derived exosomes modulate mesenchymal stem cells: potential role in leukemia progression

**DOI:** 10.1186/s12977-016-0307-4

**Published:** 2016-10-19

**Authors:** Jamal El-Saghir, Farah Nassar, Nadim Tawil, Marwan El-Sabban

**Affiliations:** 1 Department of Anatomy, Cell Biology and Physiological Sciences, Faculty of Medicine, American University of Beirut, Beirut, Lebanon; 2 Department of Internal Medicine and Experimental Pathology, Faculty of Medicine, American University of Beirut, Beirut, Lebanon; 3 Department of Immunology and Microbiology, Faculty of Medicine, American University of Beirut, Beirut, Lebanon

**Keywords:** Exosomes, Tax, Leukemia, miRNA, MSCs, NF-κB, Niche

## Abstract

**Background:**

Exosomes are membrane nano-vesicles secreted by a multitude of cells that harbor biological constituents such as proteins, lipids, mRNA and microRNA. Exosomes can potentially transfer their cargo to other cells, implicating them in many patho-physiological processes. Mesenchymal stem cells (MSCs), residents of the bone marrow and metastatic niches, potentially interact with cancer cells and/or their derived exosomes. In this study, we investigated whether exosomes derived from adult T-cell leukemia/lymphoma (ATL) cells act as intercellular messengers delivering leukemia-related genes that modulate the properties of human MSCs in favor of leukemia. We hypothesized that the cargo of ATL-derived exosomes is transferred to MSCs and alter their functional behavior to support the establishment of the appropriate microenvironment for leukemia.

**Results:**

We showed that both ATL cells (C81 and HuT-102) and patient-derived cells released Tax-containing exosomes. The cargo of HuT-102-derived exosomes consisted of miR-21, miR-155 and vascular endothelial growth factor. We demonstrated that HuT-102-derived exosomes not only deliver Tax to recipient MSCs, but also induce NF-κB activation leading to a change in cellular morphology, increase in proliferation and the induction of gene expression of migration and angiogenic markers.

**Conclusions:**

This study demonstrates that ATL-derived exosomes deliver Tax and other leukemia-related genes to MSCs and alter their properties to presumably create a more conducive milieu for leukemia. These findings highlight the contribution of leukemia-derived exosomes in cellular transformation and their potential value as biomarkers and targets in therapeutic strategies.

**Electronic supplementary material:**

The online version of this article (doi:10.1186/s12977-016-0307-4) contains supplementary material, which is available to authorized users.

## Background

Human T-cell lymphotropic virus type I (HTLV-I) is the causative agent of an aggressive CD4^+^ T cell neoplasm, adult T-cell leukemia/lymphoma (ATL) [1, 2]. Tax oncoprotein is responsible for the pathogenic potential of HTLV-I by activating the expression of viral and cellular genes involved in several pathways including nuclear factor-kappa B (NF-κB) [3–5]. NF-κB family members consist of five closely associated subunits: RelA (p65), RelB, cRel, p50 and p52 that have transactivating or transrepressing functions. NF-κB activation by Tax involves the phosphorylation of IκB-α leading to its dissociation from RelA/p50 dimer, its ubiquitination and subsequent proteasomal degradation. The heterodimer is translocated into the nucleus and activates the transcription of target genes involved mainly in cell proliferation and survival [6–10].

Exosomes are nanoscale vesicles (30–100 nm) [11, 12] derived from the endosomes of multivesicular bodies that fuse with the plasma membrane leading to their release into the extracellular milieu. They contain common proteins of the endosomal pathway and specific proteins that can be traced back to the cell of origin. Exosomes are released in vitro by multiple cell types including epithelial cells, neuronal cells, adipocytes, immune cells, fibroblasts and cancer cells, implicating them in intercellular communication. Exosomes are detected in vivo in most body fluids such as blood, urine, saliva, breast milk, amniotic fluid, ascites, synovial fluid, cerebrospinal fluid and semen, where they have the potential to be used as biomarkers [13–15]. The importance of exosomes is attributed to their roles in cell–cell communication through the delivery of signaling cues to target cells at distant sites, contributing to patho-physiological functions such as immune regulation, programmed cell death, angiogenesis, inflammation, viral infection and cancer [15–18]. Whereas the role of exosomes derived from solid cancers has been extensively investigated in supporting tumor growth, angiogenesis, metastasis and drug resistance [17, 19], their function in hematological malignancies has not been fully assessed. Some studies highlighted their immuno-suppressive functions [20–23] whereas others reported an enhanced cytotoxic effect of immune cells pre-exposed to leukemia-derived exosomes [20, 24–26]. In addition, many studies have focused on the interaction between leukemic cells and endothelial cells via exosomes. Taverna et al. demonstrated that exosomes from chronic myeloid leukemia (CML) cell lines trigger endothelial cell activation and angiogenesis due to IL-8-mediated increase in cell adhesion molecules [27, 28]. This angiogenic effect was attributed by other studies to specific exosomal microRNAs (miRNAs) that stimulate endothelial cell migration and tube formation [29, 30]. The importance of exosomal miRNAs is that they can freely shuttle between cells and alter cellular pathways involved in disease pathogenesis [31]. Distinct miRNA signature was attributed to many leukemia types including CML, acute myeloid leukemia (AML) and chronic lymphocytic leukemia (CLL) [32–34]. Recent data examined the role of leukemia-derived exosomes in delivering signaling molecules that modulate the surrounding stromal cells to further support leukemia progression [34, 35]. Moreover, exosomes from HTLV-I positive cells were found to contain viral proteins and inflammatory mediators and to protect target cells from apoptosis [36].

The area of ATL modulation of the bone marrow niche, specifically MSCs, via exosomes has not been previously explored. In this study, we investigated whether exosomes derived from ATL cells can be transferred to MSCs, residents of the bone marrow and metastatic niches, and consequently alter their functional behavior in favor of leukemia. Our data showed that ATL-derived exosomes harbor Tax, along with other leukemia-related genes, and release their cargo into MSCs. The delivery of ATL-derived exosomes led to the activation of NF-κB pathway in recipient MSCs which resulted in the induction of Tax-dependent cell proliferation and expression of migration and angiogenesis genes.

## Methods

### Reagents

DMEM (low glucose), trypsin, phosphate buffered saline (PBS), heat-inactivated fetal bovine serum (FBS), bovine serum albumin (BSA), normal goat serum (NGS), formaldehyde 37 %, PKH26 Red Fluorescent Cell Linker Kit, Arsenic trioxide (As), anti-Actin and -GAPDH antibodies were purchased from Sigma Aldrich (USA). MSCs, RPMI 1640, penicillin/streptomycin (P/S) were from Lonza (USA). Recombinant human interferon-alpha (IFN) or Roferon-A was obtained from Roche (Switzerland). Cell culture plastic ware was purchased from Corning (USA). Ultracentrifugation tubes and RevertAid 1st strand cDNA synthesis kit were from Thermo Scientific (USA). Nucleospin RNA II kit was from Macherey-Nagel (Germany). Primers were from TIB MOLBIOL (Germany). Western blot and quantitative PCR reagents were from BioRad Laboratories (USA). Luminol reagents and secondary antibodies, goat anti-mouse and goat anti-rabbit IgG-horseradish peroxidase conjugated, were purchased from Santa Cruz Biotechnology (USA). CD63 and TSG-101 antibodies were from Abcam (USA). Phospho-NF-κB p65 antibody was from Cell Signaling (USA). TaqMan^®^ MicroRNA reverse transcription kit, primers, probes and 2× universal Master Mix were purchased from Applied Biosystems (USA). VEGF enzyme-linked immunosorbent assay (ELISA) kit was from R&D systems (USA). Hoechst 33342, Prolong Anti-fade kit, CD9 antibody and Alexa 488 secondary antibody were from Life Technologies (USA). Confocal dishes were purchased from MatTek Corporation (USA). Scanning electron microscopy material was from Electron Microscopy Sciences (USA).

### Cells and culture conditions

The leukemic cell lines used in this study are either HTLV-I negative (Molt-4) or positive (C81 and HuT-102). Leukemic cell lines were maintained in RPMI 1640 medium. MSCs were maintained in DMEM low glucose medium. Media were supplemented with 10 % FBS and 1 % P/S. Patient-derived ATL cells (p-ATL) were obtained from frozen mononuclear cells of deceased ATL patients, as regulated by the American University Institutional Review Board. As previously described [37], peripheral blood from acute ATL patients was obtained and subjected to Ficoll gradient centrifugation. Mononuclear cells were collected and frozen. Primary ATL cells were thawed and used in culture experiments to collect exosomes. Cells were maintained in RPMI 1640 medium supplemented with 20 % FBS, 1 % P/S, 0.01 µg/ml of recombinant Interleukin-2 and 1 % phytohemagglutinin. All cell lines were maintained at 37 °C in a humidified incubator containing 5 % CO_2_.

### Isolation of leukemia-derived exosomes

Exosomes were prepared from culture supernatants of leukemic cells by differential centrifugations. Briefly, leukemic cell lines were maintained in their recommended media until they reach 70–80 % confluency. Then, media was replaced with serum-free media (to eliminate serum-derived exosomes) and the cells were returned to the incubator for 48–72 h. After collection, the culture supernatants were sequentially centrifuged at 300*g* for 10 min, 2000*g* for 20 min and 10,000*g* for 30 min at 4 °C to pellet cells, dead cells and cell debris, respectively. The supernatants were then filtered using a 0.22 µm filter and centrifuged at 100,000*g* for 70 min at 4 °C to pellet the exosomes using the T865 rotor in a Sorvall WX Ultra Series Floor Model Centrifuge (Thermo Scientific, USA). The exosome pellet was washed in 1 ml PBS and centrifuged again at 100,000*g* for 70 min at 4 °C using S120-AT2 rotor in a Sorvall Discovery M120 Ultracentrifuge (Thermo Scientific, USA). The final exosome pellet was re-suspended in 50 µl PBS and stored at −80 °C. A further purification step was performed using sucrose cushion. In brief, the exosome pellet was re-suspended in 25 ml PBS and was gently loaded on top of 4 ml of Tris/Sucrose/Deuterium oxide solution in AH629 swinging rotor. Following centrifugation at 100,000*g* for 70 min at 4 °C, 3.5 ml of the sucrose cushion which now contains the exosomes, were removed from the bottom of the tubes, diluted in 30 ml PBS and centrifuged again at 100,000*g* for 70 min at 4 °C. The exosome pellet was suspended in 50 µl PBS and stored at −80 °C for further experiments.

### Scanning electron microscopic characterization of leukemia-derived exosomes

The exosome pellet was fixed in 2 % paraformaldehyde and was left to adsorb on carbon adhesive tabs for 20 min in a dry environment. After one wash with PBS, the tabs were transferred to 1 % glutaraldehyde for 5 min and then washed several times in distilled water for 2 min each, for a total of eight washes. The fixed exosomes were dehydrated with an ascending gradient of ethanol (40, 60, 80 and 97 %) for 5 min per incubation. After evaporation of ethanol, the tabs were left to dry at room temperature for 24 h on a glass microscope slide. The tabs were removed with a clean forceps, mounted on aluminum specimen mounts and examined by Mira3 LM Scanning Electron Microscope (SEM) (Tescan, Czech Republic).

### Co-culture experiments

MSCs were seeded in 12 well or 6 well plates to assess proliferation or gene expression, respectively. Once cells reach 70–80 % confluency, 30 µg (for 12 well plates) or 60 µg (for 6 well plates) of purified exosomes were directly cultured with MSCs for 72 h. The amount of exosomes used to treat MSCs was comparable to other studies where it ranged between 5 µg [36, 38], 25 µg [39] and 75 µg [40] per 24 well plate. In cell proliferation studies, MSCs were either cultured alone or cultured with leukemic exosomes, with or without treatment with 1 µM of As and 1000 units/ml of IFN.

### Uptake and internalization of PKH26-labeled exosomes

Exosomes were labeled with PKH26 according to the manufacturer’s protocol with modifications. Briefly, 30 µg of HuT-102-derived exosomes were re-suspended in 1 ml of Diluent C. In another tube, 4 µl of PKH26 was mixed with 1 ml of Diluent C just prior to staining. The PKH26 suspension was mixed with the exosomes suspension and incubated for 4 min, protected from light. The staining reaction was stopped by the addition of an equal volume of 1 % BSA and centrifuged at 100,000*g* for 70 min at 4 °C. The pellet was washed with PBS and centrifuged at 100,000*g* for 70 min at 4 °C. The exosome pellet was suspended in 200 µl of supplemented DMEM low glucose medium and cultured with a confluent layer of MSCs in a confocal dish. Following co-incubation for 24 h, MSCs were washed with PBS, stained with Hoechst for 5 min, washed with PBS and fixed with 4 % formaldehyde. Images of exosome uptake by MSCs were acquired with LSM710 laser scanning microscope (Carl Zeiss, Germany).

### Cell proliferation assays

The proliferation of MSCs following co-culture with exosomes (30 µg) was assessed by Trypan Blue Exclusion assay. Cells were photographed and counted at day 3 post-co-culture. Proliferation graphs were plotted as percentage of the control, cells cultured alone, adjusted to 100 %. Each condition was counted in duplicate and three independent experiments were performed and plotted.

### Transcriptional expression analysis

Total RNA was extracted from cells or from purified exosomes using Nucleospin RNA II extraction kit as per manufacturer’s instructions. Then, cDNA was synthesized from 1 μg of total RNA using RevertAid 1st strand cDNA synthesis kit. Quantitative PCR (qPCR) was performed using iQ SYBR Green Supermix in a CFX96 system (BioRad Laboratories, USA). PCR parameters consisted of a pre-cycle of 95 °C for 3 min followed by 40 cycles consisting of 95 °C for 10 s, x °C for 30 s, and 72 °C for 30 s (x being the annealing temperature for each set of primers listed in Table 1). A final extension at 72 °C for 5 min was then performed followed by a melting curve, 55 °C, with the temperature gradually increased (0.5 °C) to 95 °C. The fluorescence quantitative cycle (Cq) value was obtained for each gene and normalized to that of the housekeeping gene, GAPDH. The comparative quantitative cycle (ΔΔCq) method was used to describe the fold change in expression of the target gene in cells retrieved from co-cultures to the same cells cultured alone. Three independent experiments were performed and presented.

**Table 1:** Primers sequence for qPCR

Primer	Sequence	Annealing temperature
VEGF	F: AGGCCCACAGGGATTTTCTT	55
	R: ATCAAACCTCACCAAGGCCA	
Tax	F: CGGATACCCAGTCTACGTGT	62
	R: GAGCCGATAACGCGTCCATCG	
CXCR4	F: CCTCCTGCTGACTATTCCCGA	55
	R: GGAACACAACCACCCACAAGT	
Nanog	F: CAGAAGGCCTCAGCACCTAC	58
	R: ATTGTTCCAGGTCTGGTTGC	
MMP-9	F: TTGACAGCGACAAGAAGTGG	55
	R: GCCATTCACGTCGTCCTTAT	
N-Cadherin	F: GGTGGAGGAGAAGAAGACCAG	58
	R: GGCATCAGGCTCCACAGT	
α-SMA	F: GACAGCTACGTGGGTGACGAA	58
	R: TTTTCCATGTCGTCCCAGTTG	
GAPDH	F: TGGTGCTCAGTGTAGCCCAG	58
	R: GGACCTGACCTGCCGTCTAG	

### miRNA analysis

Following total RNA extraction, miRNA expression profile was performed. A reverse transcription step was first performed on 10 ng of total RNA using a multiplex cDNA master mix containing 3 of the primers of interest (miR-21, miR-155 and U6B). The PCR protocol applied was at 16 °C for 30 min, then 42 °C for 30 min followed by 85 °C for 5 min, using I cycler PCR machine (BioRad Laboratories, USA). The cDNA samples were then subjected to a qPCR using specific TaqMan probes for miR-21, miR-155 and U6B. The qPCR protocol comprised the following steps: 95 °C for 10 min followed by 40 cycles of 95 °C for 15 s and finally 60 °C for 60 s. qPCR was performed in a CFX96 system (BioRad Laboratories, USA). The Cq value was obtained for each miRNA and normalized to that of the internal control, U6B. The ΔΔCq method was used to describe the fold change in expression of the target miRNA in the exosomes relative to their derived cell lines. Three independent experiments were performed and presented.

### Protein expression analysis

Cells and their purified exosomes were solubilized in lysis buffer consisting of 0.125 M Tris–HCl (pH 6.8), 2 % sodium dodecyl sulfate and 10 % glycerol. After quantification using BioRad DC protein assay kit, protein lysates (15 µg of exosomes and 100 µg of cells) were separated on 10 % SDS-PAGE gel under reducing conditions (Non-reducing conditions for CD9 and CD63 antibodies). Proteins were transferred onto polyvinylidene difluoride membranes which were blocked in 5 % skimmed milk in 0.05 % Tween-PBS. The blots were incubated with specific primary and secondary antibodies. Protein bands were detected by chemiluminescence using ChemiDoc MP Imaging System (BioRad Laboratories, USA). For quantification of the results, the blots were taken at different exposures, within linear detection range, and protein band densities were analyzed by Image J software after normalization to the housekeeping gene.

### Immunofluorescence staining

MSCs were cultured, alone or with HuT-102 exosomes in confocal dishes for 72 h, fixed with ice-cold methanol and stored at −20 °C. Cells were then washed 3 times with PBS and blocked with 5 % NGS in PBS for 1 h in a humidified chamber. Cells were incubated with primary antibody for phospho-NF-κB p65 overnight at 4 °C, followed by washing and incubation with IgG-conjugated AlexaFluor488 secondary antibody (1 µg/ml) for 1 h. The cells were then stained with Hoechst (1 µg/ml), mounted on microscopic slides using Prolong Anti-Fade and observed by LSM710 laser scanning microscope.

### Enzyme-linked immunosorbent assay

The levels of soluble VEGF were measured in HuT-102 cell-free supernatant harvested after 72 h of culture or in their derived exosomes. VEGF levels were measured by enzyme-linked immunosorbent assay (ELISA) kit, as recommended by the manufacturer’s instructions. VEGF levels were calculated from duplicate measurements and were expressed as concentration after normalization to the total amount of protein in each sample.

### Statistical analysis

Statistical significance between different conditions was determined using Student’s t test.

## Results

### Exosomes derived from ATL cells harbor Tax, miR-21, miR-155 and VEGF

**Fig. 1:**
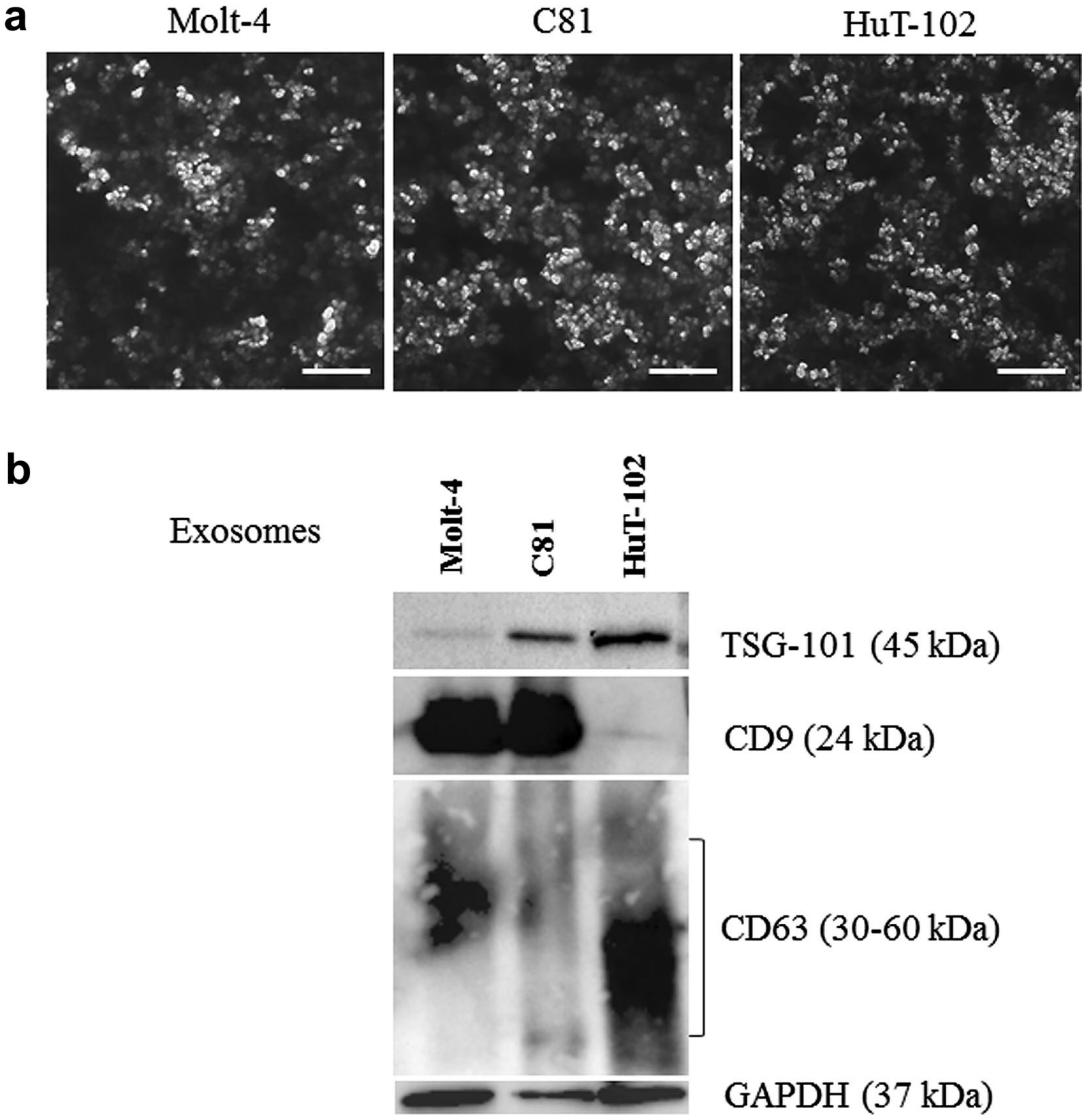
Characterization of exosomes derived from leukemic cells. **a** Scanning electron micrograph of exosomes isolated from Molt-4, C81 and HuT-102 cells, *scale bar* 1 µm. **b** A representative western blot showing the protein expression of TSG-101, CD9, CD63 and GAPDH in the lysates of exosomes (15 µg) isolated from leukemic cells

The presence of secreted exosomes in the culture supernatants of HTLV-I negative and positive cell lines was assessed by SEM and by the expression of exosome specific markers. The morphology and integrity of the isolated exosomes was first examined by SEM. The exosomes derived from the three cell lines, Molt-4, C81 and HuT-102, had similar morphological characteristics having a round shape and ranging in size from 50 to 100 nm (Fig. 1a). The identity of the purified exosomes was confirmed by immuno-blotting for specific exosome markers. TSG-101, CD9 and CD63 were expressed in the exosomes isolated from Molt-4, C81 and HuT-102 cells where CD63 appeared as a smeared pattern between 30–60 kDa (Fig. 1b).

**Fig. 2:**
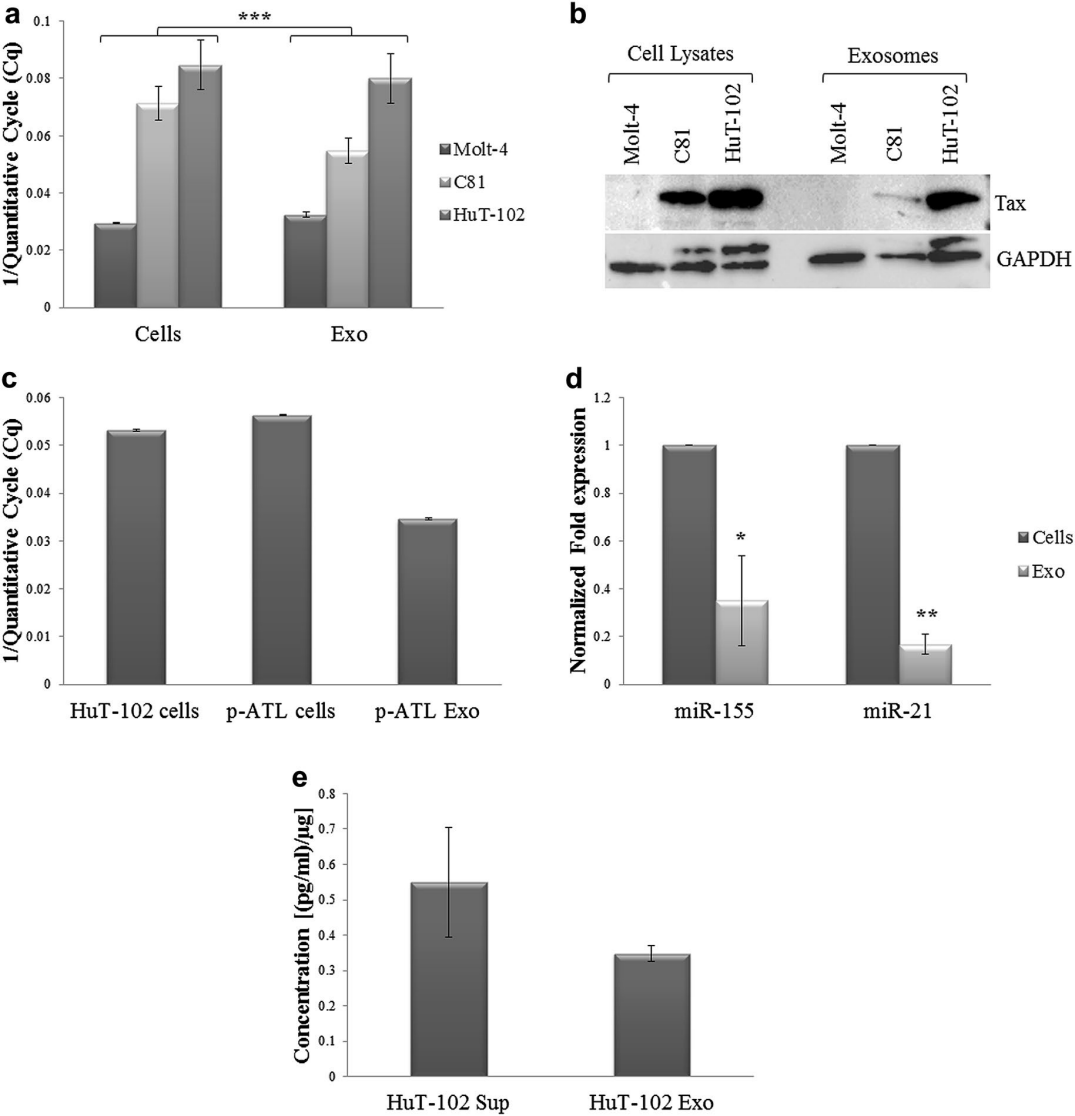
Detection of Tax, miR21, miR155 and VEGF in ATL-derived exosomes. **a** *Histogram* representing the reciprocal value of Cq at which Tax expression was detected in leukemic cells and their derived exosomes (***p ≤ 0.005). **b** A representative western blot showing Tax protein expression in leukemic cell lysates (100 µg) and their derived exosomes (15 µg). GAPDH was used as an internal loading control for the cell lysates. **c** *Histogram* representing the reciprocal value of Cq at which Tax expression was detected in p-ATL cells and their exosomes, as compared to HuT-102 cells. **d** *Histogram* representing the normalized expression of miR-155 and miR-21 in HuT-102 cells and their derived-exosomes, as detected by qPCR (*p ≤ 0.05; **p ≤ 0.01). **e** *Histogram* representing VEGF protein levels detected in the supernatants or exosomes isolated from HuT-102 cells by ELISA

The cargo of leukemia-derived exosomes included Tax which was detected in the exosomes isolated from HTLV-I positive cells and not in those isolated from negative cells, at the RNA and protein levels. Tax expression levels were comparable to that of the cells from which the exosomes were derived (Fig. 2a, b). We further demonstrated the presence of Tax mRNA in p-ATL cells and their derived exosomes (Fig. 2c). We investigated the expression of two miRNAs, miR-21 and miR-155, in HuT-102 cells and their derived exosomes, since both miRNAs were reported to be induced in HTLV-I positive cells [41, 42]. Interestingly, we detected miR-21 and miR-155 expression not only in HuT-102 cells but also in their derived exosomes (Fig. 2d). Both miRNAs were barely detectable in Molt-4 cells and exosomes while they were detected in C81 cells and exosomes at levels lower than those in HuT-102 cells and their derived exosomes (Additional file 1: Fig. S1). The protein levels of VEGF, a pro-angiogenic molecule, are found either free in the supernatants of cultured cells or associated with their derived exosomes. We detected VEGF secretion levels in HuT-102-derived exosomes at concentrations comparable to those in HuT-102 cell culture supernatants (Fig. 2e).

### Activation of NF-κB pathway in MSCs by ATL-derived exosomes

**Fig. 3:**
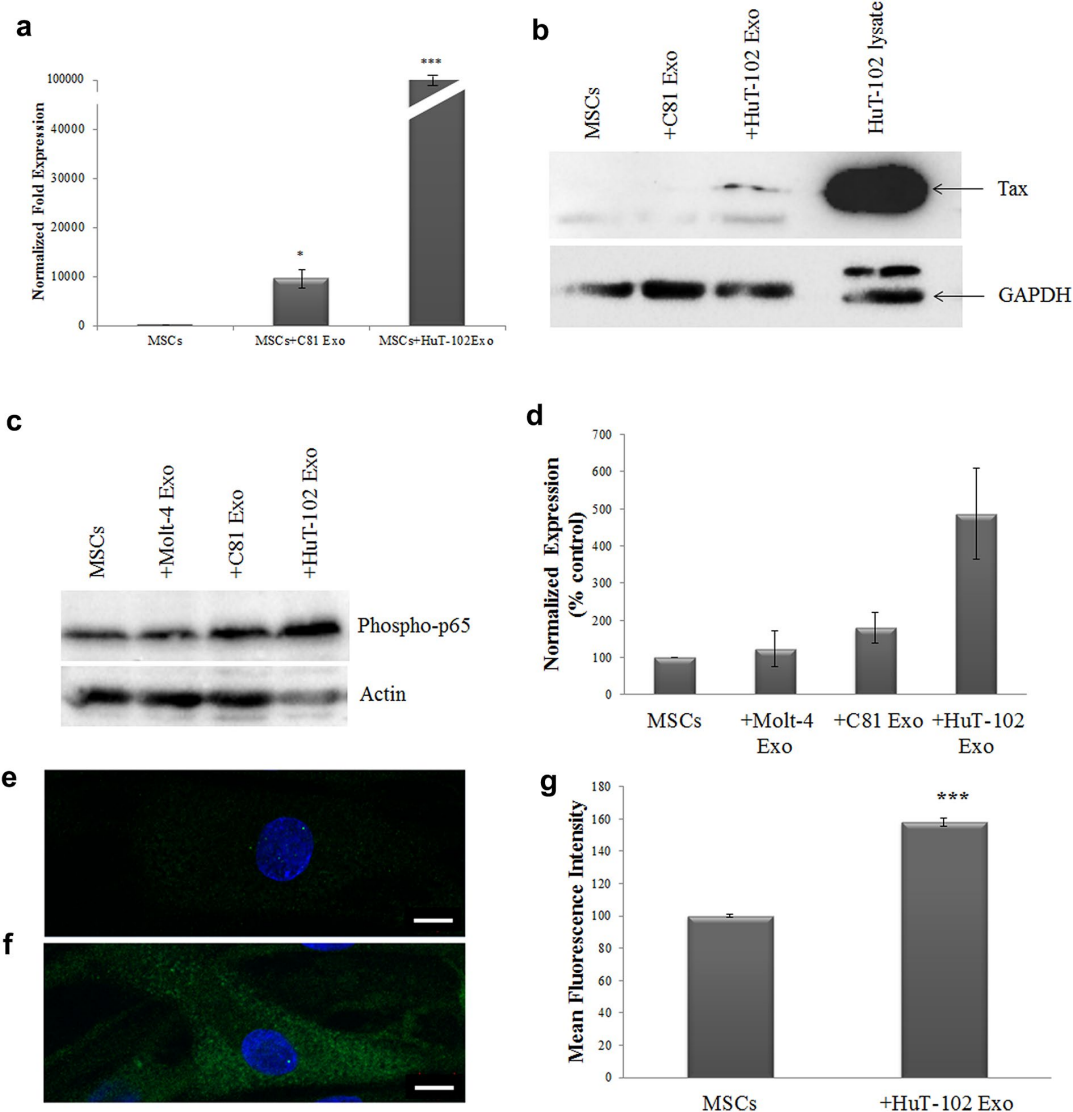
Activation of NF-κB pathway in MSCs by ATL-derived exosomes. **a** *Histogram* representing the normalized fold expression of Tax in MSCs following co-culture with C81- or HuT-102-derived exosomes, as detected by qPCR (*p < 0.05; ***p ≤ 0.005). **b** Representative western blot of Tax expression in MSCS recipient of C81 or HuT-102 exosomes, as compared to MSCs control cells. **c** Representative western blots of phospho-p65 expression in MSCs control or co-cultured with Molt-4, C81 and HuT-102-derived exosomes. Actin was used as an internal loading control. **d** Densitometry analysis of phospho-p65 protein expression in recipient MSCs from two independent western blot experiments. **e**, **f** Fluorescence images of phospho-p65 expression in MSCs control or co-cultured with HuT-102 exosomes, respectively. **g** *Histogram* representing the quantification of the mean fluorescence intensity of phospho-p65 expression in MSCs, control or co-cultured with HuT-102 exosomes, in six different fields acquired by LSM710 laser scanning microscope using a 63× objective lens, *scale bar* 10 µm (*p < 0.05)

To explore whether Tax can be delivered from HuT-102 cells to normal recipient cells via exosomes, we co-cultured HuT-102-derived exosomes with MSCs. MSCs were chosen as recipient cells since they are the residents of the bone marrow niche and the microenvironment of metastatic sites where they potentially interact with leukemic cells and/or their derived exosomes. Interestingly, significant levels of Tax mRNA were detected in MSCs recipient of HuT-102-derived exosomes, as compared to control MSCs. To provide evidence that Tax is delivered through exosomes and not through viral particles, we isolated exosomes from C81 cells, an HTLV-I transformed cell line but defective in virion production. Tax mRNA expression was detected at significant levels in MSCs following co-culture with C81 exosomes, yet at lower levels than those detected in MSCs co-cultured with HuT-102 exosomes (Fig. 3a). At the protein level, Tax expression was found at low levels in MSCs recipient of HuT-102-derived exosomes (Fig. 3b).

We then examined whether ATL-derived exosomes can activate NF-κB pathway, the main target of Tax oncoprotein [43], in MSCs. Interestingly, the expression of phospho-p65, the phosphorylated form of NF-κB subunit p65, was induced in MSCs recipient of C81 and HuT-102 exosomes by two and five fold, respectively, as compared to MSCs control or recipient of Molt-4 exosomes (Fig. 3c, d). This finding was further confirmed by immunofluorescence where a significant increase in phospho-p65 staining was observed when MSCs received HuT-102 exosomes (Fig. 3e–g). In addition, NF-κB p50 and p65 subunits were both induced in MSCs following co-culture with HuT-102-derived exosomes (Additional file 2: Fig. S2).

### ATL-derived exosomes induce MSCs proliferation and expression of NF-κB target genes

**Fig. 4:**
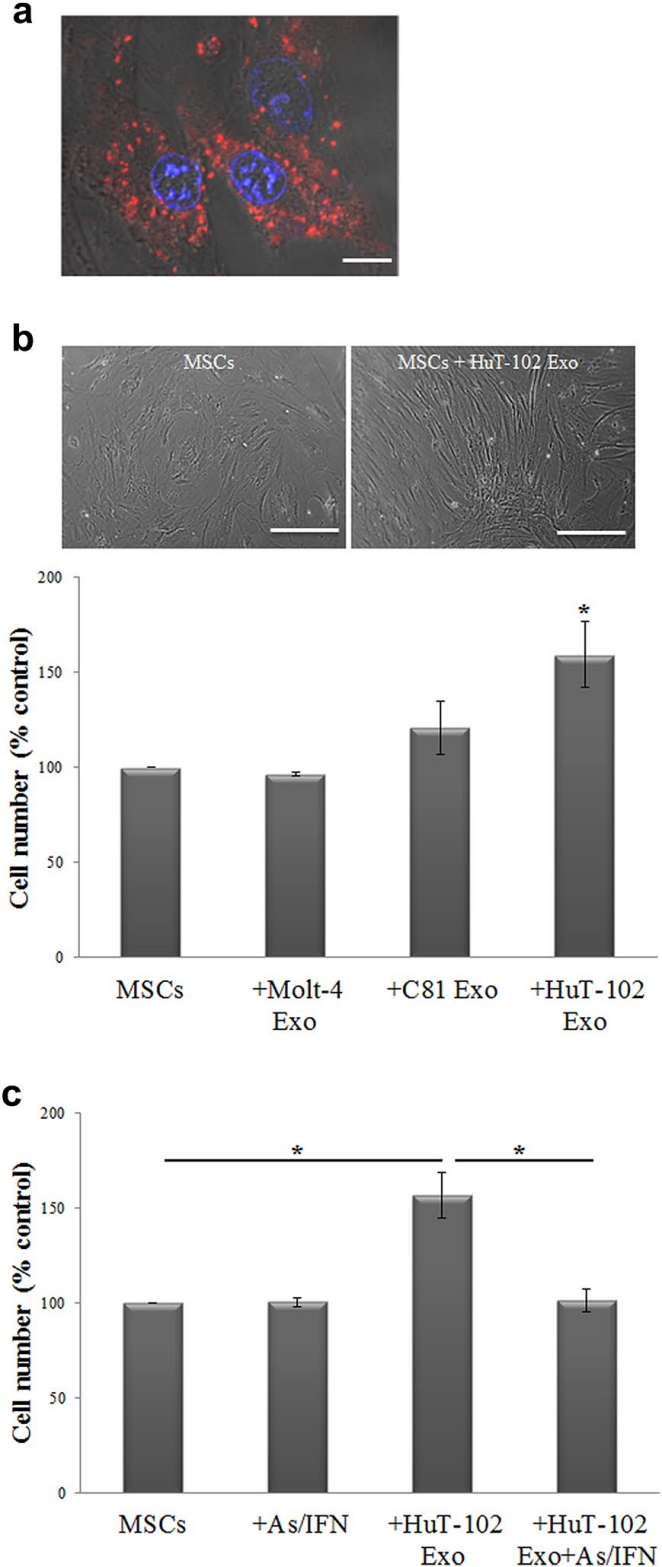
Induction of MSCs proliferation by ATL-derived exosomes. **a** Fluorescence images of MSCs following the uptake of PKH26-labeled HuT-102 exosomes (*red color*), *scale bar* 10 µm. Hoechst is used as a nuclear stain (*blue color*). **b** Bright field images of MSCs cultured alone or with 30 µg of HuT-102-derived exosomes for 72 h, *scale bar* 40 µm. *Histogram* representing the proliferation of MSCs after co-culture with leukemia-derived exosomes for 72 h (*p < 0.05). **c** *Histogram* representing the proliferation of MSCs after co-culture with HuT-102-derived exosomes for 72 h, with or without As/IFN treatment (*p < 0.05)

One of the main targets of Tax is the activation of NF-κB pathway which induces cell proliferation and transformation [43–45]. In fact, targeting HTLV-I transformed cells by the combinational treatment of As and IFN resulted in Tax down-regulation and cell death induction [46, 47]. To prove that Tax-containing exosomes can modulate MSCs proliferative properties, we first demonstrated that exosomes are indeed up taken by MSCs where PKH26-labeled exosomes from HuT-102 cells were successfully internalized following co-culture for 24 h (Fig. 4a). Upon entry of HuT-102-derived exosomes, which contained higher levels of Tax mRNA and protein than C81 exosomes (Fig. 2a, b), MSCs proliferation was greatly affected where their confluency was clearly higher and cell proliferation was induced by 60 % (Fig. 4b), as compared to cells cultured alone. This surge in proliferation was proven to be Tax-dependent since the combinational treatment of As and IFN reverted back the proliferative ability of MSCs recipient of HuT-102-derived exosomes to reach that of MSCs control (Fig. 4c).

**Fig. 5:**
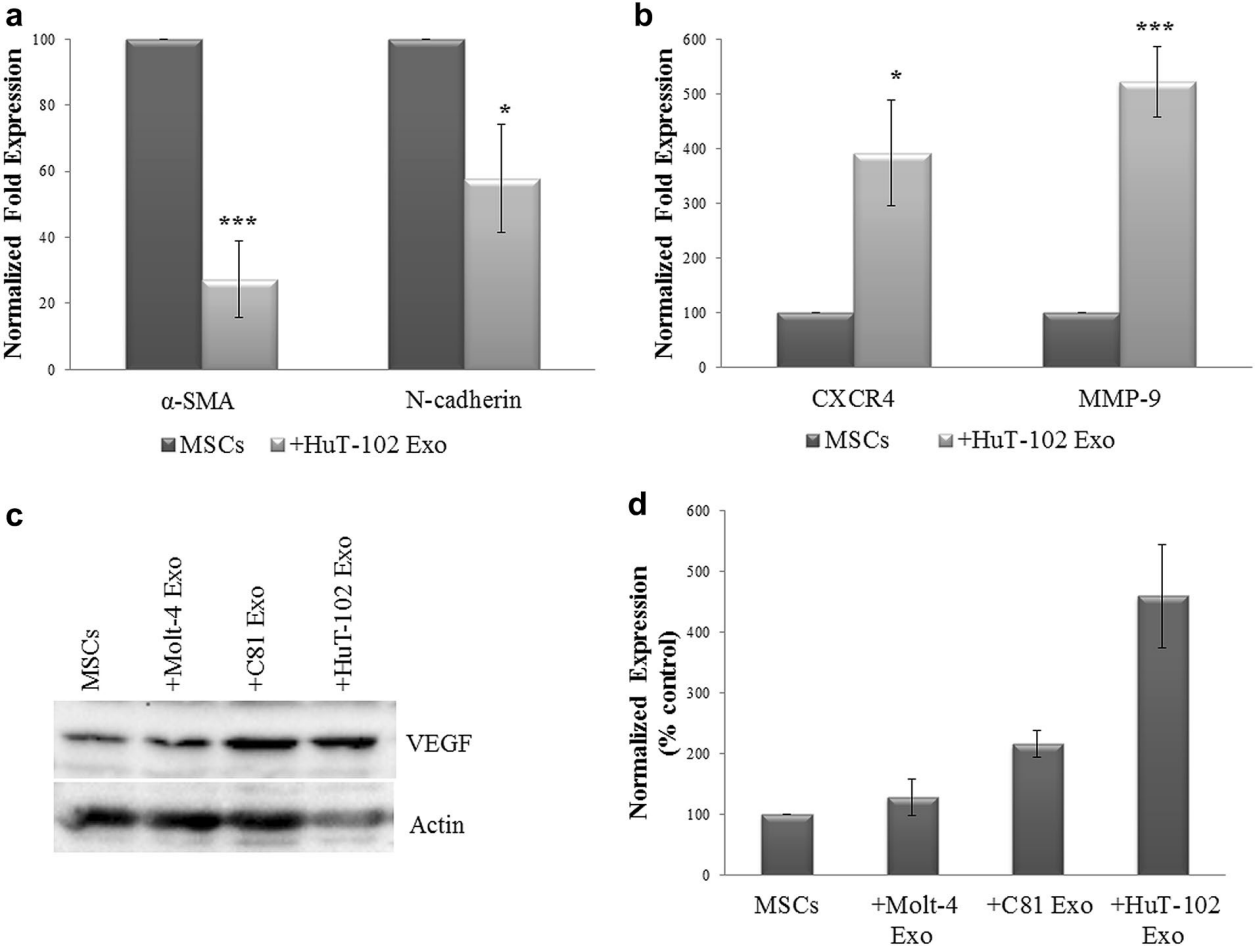
Activation of NF-κB target genes in MSCs by ATL-derived exosomes. **a**, **b** *Histograms* representing the normalized fold expression of α-SMA, N-cadherin, CXCR4 and MMP-9 in MSCs control or co-cultured with HuT-102-derived exosomes, as detected by qPCR (*p < 0.05; ***p < 0.005). **c** Representative western blots of VEGF expression in MSCs control or co-cultured with Molt-4, C81 and HuT-102-derived exosomes. Actin was used as an internal loading control. **d** *Histogram* representing the densitometry analysis of VEGF protein expression from two independent western blot experiments

The activation of NF-κB pathway involves an array of target genes involved in cell proliferation, migration and angiogenesis. Therefore, we checked the expression of stemness markers and migration genes in MSCs modulated by HuT-102 exosomes. At the transcriptional level, the expression of stemness markers alpha-smooth muscle actin (α-SMA) and N-cadherin was significantly decreased in MSCs modulated by HuT-102-derived exosomes (Fig. 5a). Although Nanog expression levels did not reach statistical significance, a decrease in mRNA levels was observed in MSCs co-cultured with exosomes harboring Tax. Furthermore, mRNA expression of CXCR4 and MMP-9 migration markers was significantly up-regulated in MSCs recipient of HuT-102 exosomes (Fig. 5b). The protein expression of VEGF, one of NF-κB target genes [48], was also increased in MSCs modulated by C81- and HuT-102-derived exosomes, as compared to MSCs control or MSCs modulated by Molt-4 exosomes (Fig. 5c, d).

## Discussion

Although HTLV-I transmission modes are well established, we hypothesize a novel infection method via exosomes whereby the main oncoprotein Tax can be delivered to normal recipient cells and subsequently activates NF-κB pathway involved in cellular transformation. The suggested infection via ATL-derived exosomes is even more effective since they can target a variety of cell lines in contrast to HTLV-I infection which specifically targets T cells [1, 2]. Since the role of exosomes in HTLV-I modulation of MSCs, residents of the bone marrow and metastatic niches, is not yet explored, we investigated whether exosomes derived from HTLV-I-infected cells can alter MSCs properties in favor of leukemia.

After the physical and molecular characterization of collected exosomes, we detected the expression of Tax in the exosomes of C81 and HuT-102 cells but not in those from Molt-4 cells, at the transcriptional and translational levels. Interestingly, we detected Tax mRNA in the exosomes isolated from p-ATL cells which underscores the in vivo relevance of such finding. Tax protein expression was previously reported in HTLV-I positive cell lines [36]. Our study is the first to detect the expression of two miRNAs, miR-21 and miR-155 in HTLV-I-derived exosomes. In fact, both microRNAs are up-regulated in HTLV-I/Tax positive cells. They enhance leukemic cell proliferation by suppressing the expression of proteins regulating p53-dependent cell death and proteins involved in inhibition of cell proliferation. In particular, miR-155 has an additive value to the HTLV-I exosomal cargo due to its close association with Tax where miR-155 was found to be induced by Tax through NF-κB activation of its promoter [41, 42]. We also revealed the existence of a pro-angiogenic molecule, VEGF, in the exosomes released by HuT-102 cells. ELISA results showed high detection levels of VEGF protein in HuT-102-derived exosomes as compared to HuT-102 supernatants. Only two studies checked for the presence of VEGF in cancer-exosomes but the obtained data was based on solid and not hematological cancers. The first one reported the transcripts of VEGF A and C in the exosomes of a rat pancreatic adenocarcinoma cell line [49]. Another group showed that VEGF, IL-6 and MMP-2 proteins were present in the culture supernatants of human melanoma cells with no direct evidence of VEGF detection in their derived exosomes [50].

We then investigated whether ATL-derived exosomes can be transferred to normal recipient cells and consequently modulate their properties. Our rationale for the choice of MSCs as target cells is that they are in vivo the resident cells of the bone marrow niche and the metastatic niches [51], where MSCs potentially interact with leukemic cells. Following direct co-culture experiments, HuT-102-derived exosomes were able to transfer Tax mRNA to recipient MSCs. Although the transfer of Tax to target cells could be potentially mediated by viral particles, we showed that exosomes from C81 cells, an HTLV-I transformed cell line but defective in virion production, harbored Tax mRNA and protein, and conveyed Tax to MSCs. The delivery of Tax to target cells via exosomes was studied by another group, however the recipient cells were either HTLV-I negative cells (Jurkat), cytotoxic T cells (CTLL-2) or peripheral blood mononuclear cells (PBMCs) [36, 52], all of which are a direct source of T cells (60–85 % in PBMCs [52]), the natural targets for HTLV-I transformation.

Since NF-κB is one of the targeted pathways by Tax [4, 5], we examined if Tax-containing exosomes can induce NF-κB activation in MSCs, our target cells. In fact, we showed that phospho-p65 subunit is up-regulated in MSCs recipient of ATL-derived exosomes. Studies have shown that the phosphorylation of NF-κB subunit p65, specifically at Serine 536, enhances p65 transcriptional activity [53]. We then investigated the downstream effects of NF-κB activation by Tax-exosomes by assessing MSCs proliferation and gene expression profile. We first showed that HuT-102-derived exosomes are certainly up taken and internalized by MSCs, as shown by the detection of PKH26-labeled exosomes inside MSCs. We then demonstrated that the delivery of Hut-102-derived exosomes, which contained high levels of Tax mRNA and protein, significantly induced MSCs’ proliferation by 60 % increase which was also evident in the enhanced confluency of the cells. This surge in proliferation was reduced with As/IFN combinational treatment further proving that the effect is Tax-dependent, even though the effect of other players, identified or not, in ATL-exosomes should not be overlooked. Tax-containing exosomes were shown to support proliferation and survival of cytotoxic T cells and PBMCs [36]. In addition, the expression of stemness markers such as α-SMA and N-cadherin expression were significantly decreased in MSCs recipient of HuT-102 exosomes. On the other hand, the expression of angiogenic and migration markers such as VEGF, CXCR4 and MMP-9 was induced in MSCs following interaction with HuT-102-derived exosomes. This data suggests that ATL-derived exosomes modulate MSCs properties causing them to lose their stemness properties in favor of a migratory and angiogenic phenotype that might support leukemia progression. Induction of VEGF and MMP expression is in accordance with other studies performed on exosomes released by solid cancers. Prostate and breast cancer-derived exosomes induced the differentiation of MSCs into myofibroblast-like cells secreting VEGF, SDF-1, TGF-β1 and MMPs, contributing to disease progression [38, 40]. As for the role of exosomes from hematological malignancies in modulating surrounding stromal cells, most of the studies focused on AML- and CML-derived exosomes promoting angiogenic ability in endothelial cells [27–30, 39].

## Conclusions

**Fig. 6:**
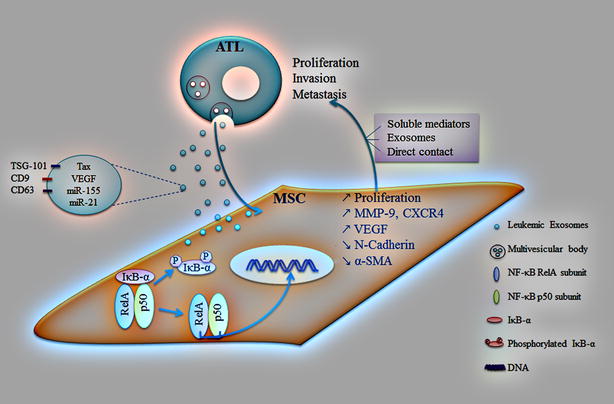
Schematic illustrating the reciprocal interaction between MSCs and ATL cells mediated by ATL-derived exosomes. Exosomes are secreted by ATL cells carrying leukemia-associated genes including Tax, VEGF and miRNAs (miR-21 and -155), in addition to exosome specific genes (TSG-101, CD9 and CD63). Upon delivery of ATL-derived exosomes to MSCs, NF-κB pathway is activated leading to IκB-α phosphorylation and subsequent degradation. The RelA/p50 dimer is now free to translocate into the nucleus and activate cellular events such as proliferation and gene expression of migration (MMP-9 and CXCR4) and angiogenic (VEGF) markers. This in turn supports ATL cells, either by paracrine interaction (soluble factors and exosomes) or by direct cell–cell contact, by creating the right microenvironment that will help ATL cells to proliferate, invade and disseminate to distant sites

This study demonstrates that Tax, along with other leukemia-related players, is delivered to MSCs via ATL-derived exosomes. This results in the activation of NF-κB pathway and its target genes involved in proliferation, migration and angiogenesis. These modulated MSCs presumably support ATL cells, either by paracrine interaction (soluble factors and exosomes) or by direct cell–cell contact, by creating a more conducive milieu for leukemia (Fig. 6). These findings highlight the contribution of leukemia-derived exosomes in cellular transformation and their potential value as biomarkers and targets in therapeutic strategies.

## Additional files

**Additional file 1: Fig. S1.** Detection of miR155 and miR21 in leukemic cells and their derived exosomes. **a**, **b** *Histograms* representing the normalized expression of miR-155 and miR-21, respectively, in Molt-4, C81 and HuT-102 cells and their derived-exosomes, as detected by qPCR.

**Additional file 2: Fig. S2.** Activation of NF-κB pathway in MSCs by Hut-102-derived exosomes. **a** Representative western blot of p50 and p65 expression in MSCs control or co-cultured with HuT-102-derived exosomes. GAPDH was used as an internal loading control. **b** *Histogram* showing densitometry analysis of p50 and p65 protein expression in MSCs control or recipient of HuT-102 exosomes, after normalization to GAPDH.

## Abbreviations

miRNA: microRNA
MSCs: mesenchymal stem cells
ATL: adult T-cell leukemia/lymphoma
VEGF: vascular endothelial growth factor
NF-κB: nuclear factor-kappa B
HTLV-I: human T-cell lymphotropic virus type I
CML: chronic myeloid leukemia
AML: acute myeloid leukemia
CLL: chronic lymphocytic leukemia
PBS: phosphate buffered saline
FBS: fetal bovine serum
NGS: normal goat serum
mRNA: messenger RNA
qPCR: quantitative polymerase chain reaction
Cq: quantitative cycle
p-ATL: patient-derived ATL cells
SEM: scanning electron microscope
IFN: recombinant human interferon-alpha
As: arsenic trioxide
CXCR4: C-X-C chemokine receptor type 4
MMP-9: matrix metalloproteinase 9
α-SMA: alpha-smooth muscle actin
TGF-β1: transforming growth factor β1
PBMCs: peripheral blood mononuclear cells
