# Supplementary figures and images for: ATL-derived exosomes modulate mesenchymal stem cells: potential role in leukemia progression
Source: Retrovirology. 2016 Oct 19;13:73. doi: 10.1186/s12977-016-0307-4 (PMC5070229; doi:10.1186/s12977-016-0307-4)

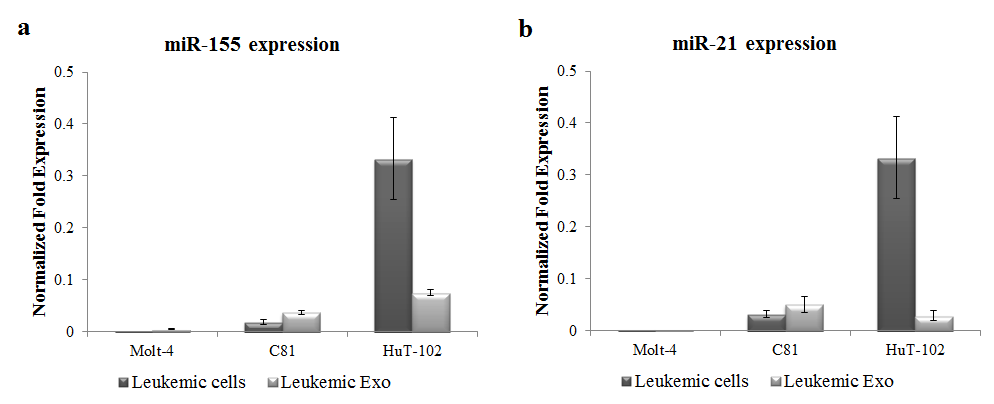

Supplement: Supplementary file 1 — Additional file 1: Fig. S1. Detection of miR155 and miR21 in leukemic cells and their derived exosomes. a, b Histograms representing the normalized expression of miR-155 and miR-21, respectively, in Molt-4, C81 and HuT-102 cells and their derived-exosomes, as detected by qPCR. [file 12977_2016_307_MOESM1_ESM.tif]

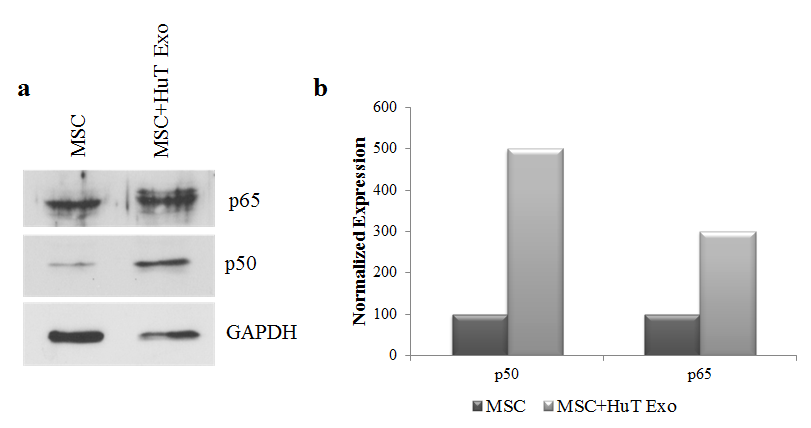

Supplement: Supplementary file 2 — Additional file 2: Fig. S2. Activation of NF-κB pathway in MSCs by Hut-102-derived exosomes. a Representative western blot of p50 and p65 expression in MSCs control or co-cultured with HuT-102-derived exosomes. GAPDH was used as an internal loading control. b Histogram showing densitometry analysis of p50 and p65 protein expression in MSCs control or recipient of HuT-102 exosomes, after normalization to GAPDH. [file 12977_2016_307_MOESM2_ESM.tif]
